# Human group size puzzle: why it is odd that we live in large societies

**DOI:** 10.1098/rsos.230559

**Published:** 2023-08-16

**Authors:** Tamas David-Barrett

**Affiliations:** University of Oxford, Trinity College, Broad Street, Oxford OX1 3BH, UK

**Keywords:** coordination, behavioural synchrony, group size, agent-based model, social networks, social technologies

## Abstract

Human groups tend to be much larger than those of non-human primates. This is a puzzle. When ecological factors do not limit primate group size, the problem of coordination creates an upper threshold even when cooperation is guaranteed. This paper offers a model of group coordination towards behavioural synchrony to spell out the mechanics of group size limits, and thus shows why it is odd that humans live in large societies. The findings suggest that many of our species' evolved social behaviours and culturally maintained social technologies emerged as solutions to this problem.

## 1. Introduction

Humans, like all non-human apes, evolved to form social groups [1,2]. Despite the similarity in many traits of social behaviour, human groups differ from non-human primate groups in several features, one of which is their size [3,4]. Yet, this observation comes with difficulties.

There have been many attempts to assess the ‘natural’ or ‘ancestral’ group size of humans, often based on forager cultures today, or the, dotted, archaeological record [5–7]. However, ecological setting rather than any inherent feature of social dynamics is often the main driver of forager group size [8–10].

The second difficulty is methodological, the core of which is the definition of a ‘human group’. Hunter–gatherers tend to self-organize into hierarchical macro-structures [11–13], which are more in line with a baboon-like troop organization than a bonobo or chimpanzee one. This is despite the fact that human micro-organization is very different from baboons, the latter being a clan, dominated by a single male, while humans form predominantly pair-bonded multi-male-multi-female societies [14].

Yet, even given these difficulties of definitions, it is clear that the natural human group [15,16], at the average of 840 individuals (defined as the highest level of the social organization, measured in 340 forager cultures [17]), is much larger than the largest coherent baboon bands at 220 individuals [18] or chimpanzee groups that are typically around 40–45 individuals, and is the largest of any non-human ape group [19,20].

Baboon bands sometimes form large meta-populations, but these are more like herds than groups in the ‘ape group’ sense [21]. Although such aggregations are rare in primates, they often occur in other mammals reaching a herd size well above the human forager average. This is especially true for ungulates: for instance, the wildebeest mega-population on the Serengeti has reached 1.2 million [22,23] or the Mongolian gazelle 200 thousand individuals [24]. Both of these ungulate species' local mega-populations form mega-herds that migrate together. In this sense, they are engaged in a form of collective action. However, the logic of these herds is predator avoidance, which is the simplest form of collective action, one that can be explained by a simple algorithm of individual physical distance management [25].

Somewhat similarly, the number of bats roosting in the same cave can range from hundreds to tens of millions [26,27]. Yet, most bats hunt alone, and even when they form foraging parties, these tend to be small groups, less than 15 in size [28].

Pair-bonded parrots and corvids, too, often form multi-male-multi-female groups, which, in the combination of these two characteristics of social organization, are similar to human groups and dissimilar to non-human primates [29–31]. However, in these, the effective foraging group size is driven by predator avoidance and not foraging ecology [32]

In ungulate herds, the primary problem is the coordination of movement [33–35], and the negotiation between conflicting interests, e.g. between large-body-sized males, and smaller females and juveniles [36,37]. However, the group's collective action's focus, i.e. the reason they are forming the group to start with, is being in each other's vicinity to reduce individual predation risk [38,39].

Compared with these non-primate examples, human groups tend to have qualitatively different tasks: doing something together, for a shared purpose, in a technology that requires complex social interaction and the maintenance of trust and cooperative stance. The full list of typical tasks that human foragers perform is dramatically different from ungulates, bats, parrots or corvids.

Most human activities are performed in small cooperative subgroups. For instance, collecting vegetal foods, shellfish, eggs, insects, small fauna or honey could be done alone as far as the technology is concerned, but tends to be a small-group activity, presumably as a predator avoidance tactic [40,41]. In this, humans are not necessarily different from non-human group-living animals. However, there are some activities for which the coordinated group action is an essential part of the technology. For instance, many methods of fishing and hunting, especially for large land mammals or large aquatic fauna, require efficient, well-coordinated collective action. No solitary human hunter could bag an elephant, or haul in a whale. The Lamalera whale hunters' example illustrates this point [42]. In this culture, people involved in fishing have two broad options: either solitary fishing for smaller catch, or collective whale hunting for a large one. There is higher average pay-off from collective whale hunting compared with solitary fishing, yet it poses a coordination problem: what we do collectively, who does what and when, rules for contingency, and principles for unexcepted events need to be coordinated beforehand among the hunters. If there are too many hands on board, the coordination efficiency falls, and the hunt is likely to be less successful [42].

These technologies not only go beyond herding or flocking behaviour in the need to rely on each other but they also tend to be more complex. Pack hunters like dolphins [43,44], wolves [45,46], African wild dogs [47] and lions [48,49] face similar group coordination problems. However, there is no known example of these packs going beyond a group size of 10–20 individuals. Among non-human primates, the chimpanzee hunt is perhaps the most similar to these predators’, which is typically also similarly sized [50–52]. At the same time, human hunting parties can reach hundreds of people in close-coordinated collective action involving many interdependent tasks and technologies, e.g. in the midwestern seasonal bison hunt [53].

The largest non-human groups are formed in times of inter-group violence, swelling up from smaller group size during peace time [54,55], and responding with temporarily changed internal group structure [56], similar to how human hunter–gatherer groups respond to similar group integration problem, but on a larger scale, during periods of war [57]. Still, even during such intensive inter-group violence periods, the group size stays smaller than humans': around 20 in hyenas, maximum 200 for chimpanzees, and a couple of hundred for baboons. Furthermore, in inter-group conflict, the warring collective action for these species includes only a subset of the population, and hence the coordinated group in these is much smaller than the numbers corresponding to group size [55,58].

Opposed to non-human animals, human populations regularly, and in all cultures, engage in complex group activities that require solving complicated coordination problems, and are not seen in any non-human animal [59,60], but including the Neanderthal line of humans [61]. These hunting, fishing, ecosystem management, and warring activities are not only complex, but they are also scalable, and often scaled in size [62,63].

In other words, human collective action in small groups can be seen as similar to some group-living animals’, although typically more complex. However, unlike in humans, these non-human examples of complex collective action always run into a group size limit, even when larger groups would be clearly advantageous, like in the case of inter-group violence. At the same time, examples of large animal groups, like ungulate herds, schooling fish, and flocking birds, follow a simple logic of collective action: that of predator avoidance. And while more complex collective action can occur in bats, parrots and corvids that, at the same time, live in large mega-populations, the logic of these super-large groups is either predator avoidance, or parallel use of a resource, like a shelter, or prey swamping, and the complex collective action, like forming foraging parties, nest building, occurs in small groups only.

Furthermore, in different technological environments, humans regularly form groups of millions and even billions of people. The median country size today is 6.7 million people, and the median size of the largest city in any one country is 2.1 million people [64]. Arguably, the global society is so interconnected [65,66] that it can be regarded as a group itself with the size of 8 billion people. (N.B. it is not clear if it is justified to count modern societies as *natural* groups. We know from other areas of human life that modernity can change our ancestral ‘setting’, for instance the majority of us gave up foraging for farming [67,68], equity for inequity [69,70], and kinship-based social networks for friendship-based ones [71–73].) Thus, not only the ‘natural’ group size is so much larger than the groups of non-human primates, but our modern societies are so enormous that there may not be a natural group size to start with.

This is a puzzle. How come that human ape groups are so large? And if they can be this large, how come that other apes' and non-ape primates’ groups are not as large? Much of the scientific literature about human social evolution has been concerned with the tricks and ruses that our species evolved and invented to allow large group size. But under these shelves of papers lies the assumption that forming super-large groups is something unnatural, something odd. If there was no limit, why would there be a need for a trick to break through it? To my knowledge, there is no paper in the literature that spells out this assumption by asking the question: why is it odd that we live in large societies?

## 2. Methods and results

Let *n* agents form a randomly connected graph of degree *k*, with adjacency matrix a = {a*_i,j_*}_I,*j*_, where a*_i,j_* = 1 if the agents *i* and *j* are connected to each other. These agents face a problem such that they must coordinate their action to be able to act as one. The coordination takes place on a unit circle, as if each task was finding a shared direction on a compass [74–77].

The agents start with a randomly assigned initial value drawn from a uniform distribution on a compass (for the reason of the choice of using synchronization on a compass, see the electronic supplementary material, discussion and figure S1).

For the group to reach behavioural synchrony, the agents go through a series of pair-wise meetings in which they synchronize their *ϕ* values. For each synchronization event, two connected agents are randomly picked, and their *ϕ* values set to the mid-point of their old *ϕ* values

$$i,j\tilde{U}\{1,\ldots ,n\}|i\neq j,a_{i,j}=1$$

and

$$\phi_{0,i}\tilde{U}(0^{\circ },360^{\circ }),$$

where

$$f1=\{\begin{array}{ccc} \frac{\phi_{t,i}+\phi_{t,j}}{2} & \mathrm{if} & |\phi_{t,i}-\phi_{t,j}|\leq 180^{\circ }\\ \frac{\phi_{t,i}+\phi_{t,j}-360^{\circ }}{2} & \mathrm{if} & \phi_{t,i}-\phi_{t,j}>180^{\circ }\\ \frac{\phi_{t,i}+\phi_{t,j}+360^{\circ }}{2} & \mathrm{if} & \phi_{t,j}-\phi_{t,i}>180^{\circ } \end{array}.$$

Let *δ* denote the average distance among the *ϕ*s of all the group members.

$$\delta_{t}=\underset{i}{\sum }\underset{i}{\sum }|\phi_{t,i}-\phi_{t,j}|,$$

where *t* is the average number of meetings an agent has, and the | | distance measure refers to the nearer side of the unit circle. (For instance, the distance between 10 and 90 is 80, while the distance between 10 and 350 is 20.) Notice that *δ* is an inverse measure of synchrony, i.e. group's coordination efficiency is high when *δ* is low, and vice versa.

Independent of network structure, as long as *k* > 2 and the graph is connected, the *ϕ* values converge and the *δ* goes to zero with *t* increasing (electronic supplementary material, figure S2). That is, as the number of meetings goes up, the group turns towards the same direction.

This pattern of convergence is not surprising. This is an instantiation of an established result in network science, often employed to describe innovation diffusion in social networks [78,79]: unless k = 2 *and* the graph is circular, convergence happens. The only question is the speed.

Notice that the speed of convergence is driven by the average number of edges per node (electronic supplementary material, figure S2). This is also not surprising: the more connected a network is, the faster the synchronization is, another established network science fact. This speed difference is important, because if the network describes a real-world human (or other species') cooperating group of individuals, then the amount of time that the group members can spend is likely to be limited by ecological, technological factors.

**Figure 1.**
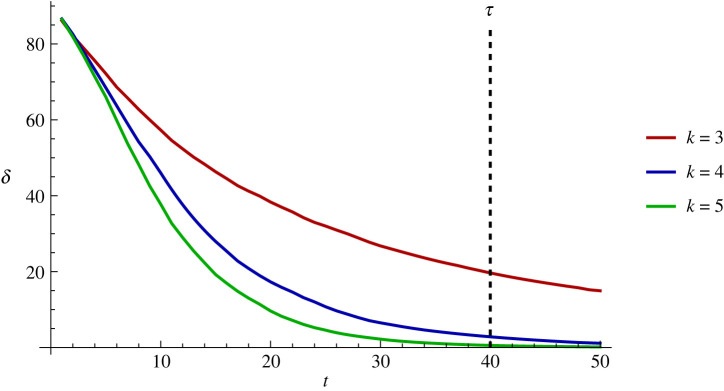
Introducing a limit to the number of meetings the agents can have illustrates the importance of the fact that the speed of convergence varies with the average degree (*n* = 20).

Let us introduce a time limit, denoted by *τ*, as a parameter external to the coordination problem (figure 1). Not surprisingly, given any arbitrary time limit, the higher the degree is, the more synchronized a group is at the cut-off point.

Thus, the time limit to coordinating the action, a constraint that all human and other animal groups face in practice, makes the degree of the social network play a key role. This raises the question of the degree's origin. Where does the number of connections per agent come from?

Let us assume that evolution (or economics) works on the level of the individuals, and thus that the agents decide their number of social connections for themselves. Let us assume that social connections come with synchronization benefits and costs of relationship maintenance that translate into a pay-off

$$p_{i}=-\overset{n}{\underset{\;j=1}{\sum }}|\delta_{i}-\delta_{j}|-\beta \cdot k_{i},$$

where *p* is pay-off or evolutionary fitness, and *β* is the cost parameter.

To evolve the optimal degree, let us use an evolutionary algorithm the following way:

Step 1: For a group of *n* agents, assign random degree uniformly to each agent such that *k_i_* denotes the degree of node *i*, with one agent having *k* − 1, and one *k* + 1 connections. (This set-up ensures that the corresponding graph exists).

Step 2: Run a large number of group coordination events, with a fixed limit of average number of meetings at *τ*. Calculate the mean pay-off for each agent.

Step 3: Given the pay-offs, set *k* to the degree number of the best performing agent.

Repeat steps 1–3 for an evolutionary round.

Using this selection algorithm, the degree evolves in line with the social costs of an edge (electronic supplementary material, figure S3). If contact maintenance costs are low, then the individuals are best off to have many social connections, and vice versa, when costs are high, the social network will be sparse.

Thus, from the fact that the group needs to coordinate its action comes two opposing pressures. On one hand, the group's coordination efficiency increases when there are more connections among the agents (electronic supplementary material, figure S2). On the other hand, the agents will end up limiting the number of connections if these are costly (electronic supplementary material, figure S3).

Notice that there are two kinds of costs in this model: one on the individual's level, and one on the group's level. The cost of an edge is suffered by the individual, and is not linked to the group's coordination problem. However, a loss of group-level coordination efficiency emerges if *k* is less than maximum.

**Figure 2.**
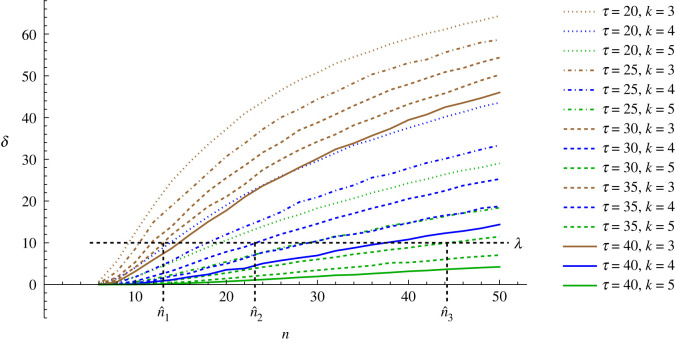
A minimum coordination efficiency threshold limits the maximum group size. The curves represent the relationship between the inverse synchronization efficiency, *δ*, and the group size, *n*, given the time limit, *τ,* and the degree, *k*, implied by the cost parameter *β*. Notice that if the efficiency threshold parameter takes the value of *λ* = 10, for instance, then this also implies a maximum group size corresponding for each *τ* and *k*. For example, for *k* = 3 and *τ* = 35 the maximum group size is ${\hat{n}}_{1}=13$, for *k* = 4 and *τ* = 30 it is ${\hat{n}}_{2}=23$, and for *k* = 4 and *τ* = 40 it is ${\hat{n}}_{3}=44$.

Notice also that given the coordination problem, the two parameters *τ* and *β* together determine the relationship between group size and synchronization efficiency (figure 2).

If the ecological or technological problem the group faces is such that there is a coordination threshold above which the group action falls apart, and under which the group operates as a unit, then this limit also serves as a maximum group size limit. Let *λ* denote such a threshold in *δ*, given the relationship between *n* and *δ*, the maximum group size is set, $\hat{n}$ .

Notice that as the *δ*(*n*) function is entirely determined by *τ* and *k*, with the latter entirely depending on *β*, and the equation $\delta (\hat{n})=\lambda$ yields $\hat{n}$, there is no wriggle room: the three parameters (*τ, β* and *λ*), and nothing else, determine the maximum group size. Thus, it is the ecological setting together with the nature of group coordination that set the maximum group size. Above this, a group cannot function in this model.

## 3. Discussion

This paper used three parameters as a frame to collective action coordination: time limit, cost of maintaining a social connection, and minimum coordination efficiency threshold. The result showed that in a behavioural synchrony framing these three parameters together determine a maximum group size.

There are well-documented human behaviours in which the need to reach behavioural synchrony efficiently limits group size. For instance, jazz jam sessions are limited to small musical band size [80–82].

Four empirical questions follow: (i) is there a group size limit for non-human primates, (ii) if so, do human groups break out of this limit, (iii) if so, are there special evolved or cultural ‘solutions’ in place to facilitate going through such a threshold, and (iv) is there a possible ‘social technologies exchange’ with eusocial insects living in complex, very large groups?

First, the group size distribution of non-human primates (electronic supplementary material, figure S4) is in line with the model's suggestion that such maximum group size exists: most primate species' group size is under 20, and among the few that have larger groups, none is above 100. Our closest relatives’, the other great apes', average group size ranges from nine (eastern gorilla), to 42–46 (chimpanzee and bonobo), with all the orangutan and gorilla species falling in the lower end of the range [19,83,84].

Second, ethnographic data are in line with the suggestion that the human group size is beyond a threshold that other primates did not cross.

For instance, let us consider the case of small languages: the median speaker number of a language is under 1000 on all continents [85,86]. The highest language diversity on the planet is on the island of New Guinea, which consists of Irian Jaya (part of Indonesia), and the mainland of Papua New Guinea. Here, the extremely high language diversity is due to the geography of the island. As the Australian continental plate pushed northward against an Easter protrusion of the Pacific plate [87], the crust of the Earth formed a mountain range with hundreds of deep valleys, almost every one corresponding to a different language [88].

In this highland region, crossing other groups’ territory is highly perilous, intergroup violence is ongoing. (For instance, in 2008, the director of the Timika hospital told me that one of the most frequent injuries they had to deal with was from arrow wounds. Similarly, when I asked tribal leaders about with whom they had conflict, they invariably mentioned nearby, competing tribes rather than the Indonesian military.) The most likely explanation for the separation of the languages is that it is impossible to maintain military domination across several valleys at the same time. As an exception that supports the point, the Dani culture, which has approximately 600 000 speakers, occupies the only large flat area of Irian Jaya Highlands: the Baliem Valley.

In the Papuan example, the median number of speakers per language, which is 643, probably corresponds not only to an ethnolinguistic meta-population, but also to a reasonable estimate of the number of people who live in a relatively small valley, relying on each other for protection against the enemies around. For this reason, 643 may be seen as a good estimate for the highest-level group size in the Highlands, whether they split into separate settlements or not. This is in line with the observation from a very early, 1961, population survey that while the average settlement size was 159–170 people, depending on measurement [89,90], the settlement sizes were driven by ecological factors, with the largest reaching above 1000 people [88]. (N.B. Irian Jaya was first entered by outsiders in the mid-1950s).

Using the New Guinea example, the average human group size is at least four times as high as that of other great apes, possibly 15 times larger. And when comparing maximum group sizes, the difference is even starker. The largest non-human great ape group ever recorded was the Ngogo chimpanzee ‘community’ that ranged between 140 and 206 members [91]. Compare that with the 1000+ settlement size of the 1961's New Guinea maximum settlement size, let alone human communities today that go into the tens of thousands to millions.

Third, how is this large human group size possible? To what extent did our species' tricks in ‘social technologies’ evolve or were invented to solve the problem of the looming group size limit? Did language evolve as a third-party information processing tool to facilitate larger groups [92–95]? Did structural solutions for macro-network management, like fission–fusion pattern, evolve or emerge to facilitate large groups or rather to facilitate temporal variation in the environment's carrying capacity [96–100]? In particular, is the presence of a baboon-like fission–fusion dynamic a clue towards the evolution of the ability to form large complex groups in humans, or is this a case of parallel evolution [14,101]? Is a central figure's one-way communication, as in the case of priesthood [102] or in social technologies facilitated by mass media [103,104], another social technology that allows larger groups?

Fourth, although the human ability to form large, complex groups is unique among vertebrates, eusocial insects tend to form hives or nests that often comprise tens of thousands of individuals. These perform a range of complex coordinating tasks like nest building, nest hunting, collective foraging [105–108], and even actively regulating the structure of social network [109]. The logic of these groups is fundamentally different from group-living mammals or birds in that in eusocial insects the individuals tend to be highly related to each other [110], and in this sense the evolution of eusocial insect is more similar to the evolution of multicellularity, in which the equivalent function to, say, an individual mammal, evolved to be present on the level of the group [111]. Nevertheless, the model presented in this paper might be general enough to explain eusocial animal cases, too, where natural selection could favour group-level benefit, a logic that might even extend to other collectively foraging insect species [112]. And, vice versa, this model might shed light to how the internal logic of solving the coordination problem in exceptionally large eusocial groups might be used for creating new social technologies for our own species.

Answering these questions is essential for understanding how it could be possible to use social technologies that would facilitate building a cohesive group of 8 billion human apes, which is arguably essential for this species to tackle the substantial coordination problems it faces on a global scale.

Note that this paper focuses on group size being limited only by coordination efficiency. This model assumes away the problem of cooperative stance, and dyadic cooperation is implied in the cost of maintaining the social network edges. Yet, there is an entire library on the origins of costly cooperation [113–120], and the interaction between network structure and cooperative stance [95,121–142], and even the possible conflict between these dynamics [143–145], represented by the negative relationship between the social cost parameter of this paper, and the maximum group size. To the extent the interaction between the coordination and cooperation dynamics shaped the evolution of network building traits is subject of future research.

## Data Availability

I have included the Wolfram Mathematica notebook that contains all the code. The data are provided in electronic supplementary material [146].
